# BAP1/ASXL1 recruitment and activation for H2A deubiquitination

**DOI:** 10.1038/ncomms10292

**Published:** 2016-01-07

**Authors:** Danny D. Sahtoe, Willem J. van Dijk, Reggy Ekkebus, Huib Ovaa, Titia K. Sixma

**Affiliations:** 1 Division of Biochemistry and Cancer Genomics Center, Netherlands Cancer Institute, Plesmanlaan 121, 1066 CX Amsterdam, The Netherlands; 2 Division of Cell Biology II, Netherlands Cancer Institute, Plesmanlaan 121, 1066CX Amsterdam, The Netherlands

## Abstract

The deubiquitinating enzyme BAP1 is an important tumor suppressor that has drawn attention in the clinic since its loss leads to a variety of cancers. BAP1 is activated by ASXL1 to deubiquitinate mono-ubiquitinated H2A at K119 in Polycomb gene repression, but the mechanism of this reaction remains poorly defined. Here we show that the BAP1 C-terminal extension is important for H2A deubiquitination by auto-recruiting BAP1 to nucleosomes in a process that does not require the nucleosome acidic patch. This initial encounter-like complex is unproductive and needs to be activated by the DEUBAD domains of ASXL1, ASXL2 or ASXL3 to increase BAP1's affinity for ubiquitin on H2A, to drive the deubiquitination reaction. The reaction is specific for Polycomb modifications of H2A as the complex cannot deubiquitinate the DNA damage-dependent ubiquitination at H2A K13/15. Our results contribute to the molecular understanding of this important tumor suppressor.

## Abstract (web summary)

The tumor suppressor BAP1 is activated by ASXL1 to deubiquitinate mono-ubiquitinated H2A at K119 in Polycomb gene repression. Here, the authors show how BAP1's C-terminal extension auto-recruits it to nucleosomes, where the DEUBAD domain of ASXL1 increases BAP1's affinity for ubiquitin to drive deubiquitination.

## Graphical abstract

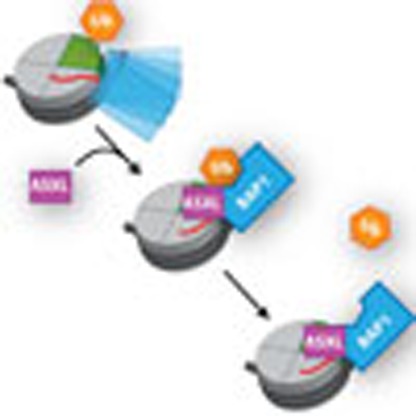

The deubiquitinating enzyme (DUB) BRCA-1-associated protein 1 (BAP1) is a critical tumor suppressor that has attracted medical interest in the past years since its loss leads to a variety of cancers^1^ including metastatic cutaneous and uveal melanoma, pleural mesothelioma, renal cell carcinoma and bladder tumours^2^,^3^,^4^,^5^,^6^,^7^,^8^. Germ-line loss of BAP1 leads to a predisposition for cancer and BAP1 tumours are associated with high tumor aggressiveness and poor prognosis^9^. The molecular pathogenesis of disorders associated with BAP1 dysfunction is poorly understood which reflects our incomplete understanding of BAP1 biology.

BAP1 has several targets and functions of which many take place in the nucleus. It participates in the DNA-damage response, DNA synthesis and cell cycle progression^10^,^11^,^12^. For many of these functions the catalytic activity of the enzyme is required. An emerging, but poorly understood role for BAP1 is in transcriptional regulation. Several studies indicate that BAP1 associates with transcription-related proteins such as FOXK2, HCF-1, OGT, LSD2, MBD-6, MBD-5, ASXL1 and ASXL2 to regulate the expression of target genes^8^,^13^,^14^,^15^.

Polycomb group proteins assemble multi-protein complexes on DNA and mediate transcriptional repression of developmental genes by compacting chromatin^16^. BAP1 is an essential part of the Polycomb repression machinery in *Drosophila*, where it deubiquitinates mono-ubiquitinated histone 2A (H2A) at lysine 118 (lysine 119 in humans), a modification that is catalysed by the Polycomb repressive complex 1 (PRC-1; refs 17, 18). For BAP1 to deubiquitinate H2A, it requires activation by an N-terminal fragment of Polycomb group protein ASX, a protein necessary for the long-term repression of HOX genes^19^. This complex is highly conserved and also deubiquitinates H2A in mammals^7^,^11^,^14^. The deubiquitination reaction was recently been shown to be augmented by loss of the C-terminal regions of human ASX (ASXL1), including its PHD finger^20^. While recent reports indicate that PRC-1-mediated H2A ubiquitination is dispensable for Polycomb repression of target genes, it is still essential for viability and H3 trimethylation by the PRC-2 (refs 21, 22).

Humans possess three homologs of *Drosophila* ASX, named additional sexcombs like 1 (ASXL1), ASXL2 and ASXL3 which are all linked to gene regulation and are often disrupted in human cancers^23^. ASXL1 disruption is especially detrimental, as it is strongly associated with acute and chronic myeloid leukaemia, chronic myelomonocytic leukaemia and other cancers^23^. ASXL1 deregulation is also linked to myelodysplastic syndrome, a disorder that conditional BAP1 knock-out mice also develop, that can lead to leukaemia^8^. In addition, ASXL1 is together with ASXL3 implicated in the Böhring–Opitz syndrome, a disorder characterized by malformation of the body and intellectual disability^24^.

BAP1 is a member of the ubiquitin C-terminal hydrolase family of DUBs, together with UCH-L1, UCH-L3 and UCH-L5. These are all cysteine proteases with a conserved catalytic UCH domain (CD). Only BAP1 and UCH-L5 share a conserved ULD domain at their C-termini. In UCH-L5 the orientation of the ULD domain is important for enzymatic activity^25^,^26^. The ULD domain in BAP1 is likely structurally related to the UCH-L5 ULD, but BAP1 contains a ∼350 amino acid (aa) insert between its CD and ULD. This insert does not resemble any known conserved domains but is nevertheless important for certain functions such as binding to the HCF-1 protein^7^,^13^.

While regulation of DUB activity by protein partners is widespread^27^,^28^,^29^, in the UCH family, only BAP1 and UCH-L5 are known to be regulated by protein partners. Phylogenetic analysis of these protein partners, ASXL1 for BAP1, and RPN13 and INO80G for UCH-L5, indicates that they all share a conserved DEUBiquitinase ADaptor (DEUBAD) domain^30^. While for UCH-L5 regulators RPN13 and INO80G the regulatory mechanisms have been described^25^,^26^, it is still unknown whether the putative ASXL1 DEUBAD domain is sufficient to regulate BAP1 and if this mechanism is related to the UCH-L5 regulatory mechanism.

Here we analyse the mechanism of H2A deubiquitination by the BAP1/ASXL1 complex. We demonstrate that the enzymatic activity is mechanistically similar to the related UCH-L5/RPN13 complex. The DEUBAD domains of ASXL1, ASXL2 and ASXL3 can activate BAP1 by increasing its affinity for ubiquitin. This by itself is not sufficient for deubiquitination of BAP1's physiological substrate mono-ubiquitinated H2A K119, but also requires the BAP1 C-terminal extension (CTE) that auto-recruits BAP1 to nucleosomes.

## Results

### ASX DEUBAD domains activate BAP1 on H2A K119-Ub

**Figure 1:**
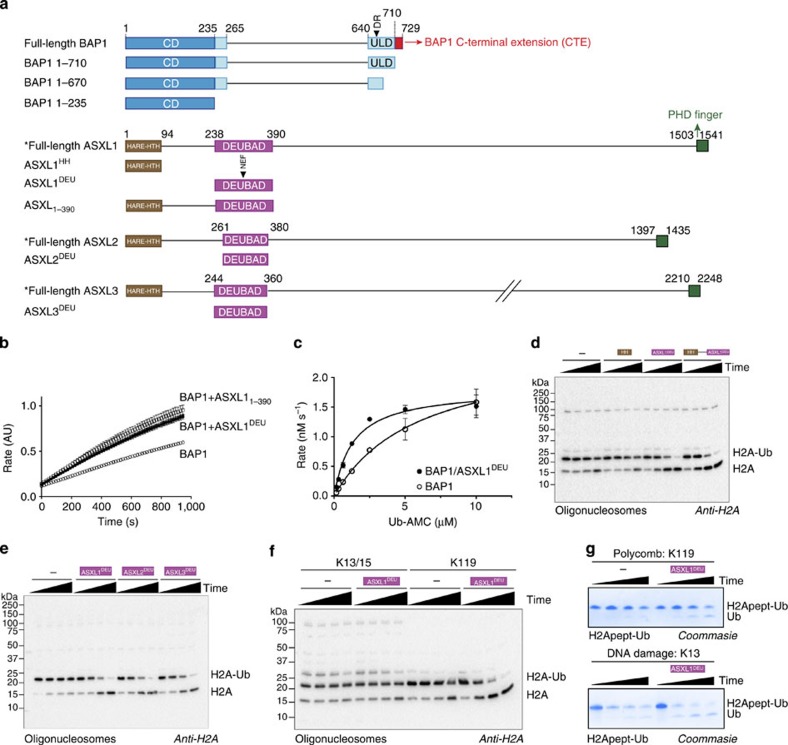
The DEUBAD domain in ASXL proteins activates BAP1 to specifically deubiquitinate K119 on H2A. (**a**) Overview of constructs. Locations of point mutations indicated with filled triangles. The asterisk highlights constructs not used in this study. (**b**) The DEUBAD domain but not the HARE-HTH domain of ASXL1 can stimulate BAP1 activity in an assay against minimal substrate Ub-AMC. Error bars, s.d. (*n*=3 independent experiments). (**c**) In Ub-AMC Michaelis–Menten analysis the BAP1/ASXL1^DEU^ complex (closed circles) has a lower K_M_ than BAP1 alone (open circles). Error bars, s.d. (*n*=3 independent experiments). (**d**) Anti-H2A western blot showing that the DEUBAD domain but not the HARE-HTH domain of ASXL1^DEU^ can stimulate BAP1 activity in a deubiquitination assay against K119 mono-ubiquitinated H2A in oligonucleosomes (time points: 0, 1, 5, 25 min). BAP1 alone reactions are indicated with ‘−'. (**e**) The DEUBAD domains of ASXL2 and ASXL3 can also activate BAP1 deubiquitination of oligonucleosomal H2A as shown in an anti-H2A western blot. (**f**) An anti-H2A western blot shows that the BAP1/ASXL1^DEU^ complex has low activity against H2A mono-ubiquitinated at K13/15 in oligonucleosomes, compared with H2A K119 ubiquitinated oligonucleosomes (time points: 0, 1, 5, 25 min). (**g**) On minimal peptide substrates derived from H2A, where either K13 or K119 are ubiquitinated chemically, the BAP1/ASXL1^DEU^ complex can deubiquitinate K13-linked ubiquitin as efficiently as K119-linked ubiquitin as visualized on SDS-PAGE coomassie.

The N-terminus of ASXL1 is responsible for BAP1 activation^17^. This region contains a predicted HARE-HTH DNA-binding domain and a DEUBAD domain (Fig. 1a)^30^,^31^. We expressed and purified constructs of these domains (Supplementary Fig. 1a) and tested what their contribution was to BAP1 activation *in vitro* using the minimal DUB substrate ubiquitin-7-amido-4-methylcoumarin (Ub-AMC)^32^. We found that ASXL1_1–390_ could activate the reaction (Fig. 1b). Within this region most of the activity is present in the ASXL1 DEUBAD domain (ASXL1^DEU^), since a construct (238–390), lacking the HARE-HTH domain (ASXL1^HH^), could activate BAP1 to the same extent (Fig. 1b). Enzyme kinetics analysis indicated that the activation effect of ASXL1^DEU^ is modest on this artificial substrate, improving *K*_*M*_ 4-fold with a minor effect on *k*_cat_ (Fig. 1c and Supplementary Table 1).

To investigate BAP1 activity on a relevant substrate, we mono-ubiquitinated H2A at K119 either in purified oligonucleosomes (Supplementary Fig. 1b) or in recombinant nucleosome core particles (NCPs). As E3 ligase we used RING1B/MI1, one of the possible catalytic modules for PRC-1 complexes that has high selectivity for K119. Like on Ub-AMC, ASXL1_1–390_ could activate BAP1 to the same extent as ASXL1^DEU^ (Fig. 1d), but on the nucleosomal substrates the activation observed was much more significant than on the minimal substrate.

The ASXL1^HH^ domain hardly changed BAP1 activity on Ub-AMC, but since this ASXL1^HH^ construct contains a putative DNA-binding domain, we tested if it was active on oligonucleosomal H2A. This was not the case; the ASXL1^HH^ construct had no effect on H2A deubiquitination, confirming that the ASXL1-dependent activation effect is fully contained in the DEUBAD domain (Fig. 1d and Supplementary Fig. 1c).

ASXL2 and ASXL3 also contain predicted DEUBAD domains (Supplementary Fig. 1d), with >60% sequence identity to ASXL1. Proteomics analyses^8^,^33^ have observed interaction of BAP1 with ASXL2^DEU^, but not yet with ASXL3^DEU^. We tested whether the ASXL2 and ASXL3 DEUBAD domains could also stimulate H2A deubiquitination by BAP1. This was the case for both ASXL2^DEU^ and ASXL3^DEU^, with kinetics comparable to ASXL1^DEU^ (Fig. 1e). These results establish that the DEUBAD domains in all human ASXL paralogs can stimulate BAP1 activity.

### The BAP1/ASXL1 complex is specific for the Polycomb site

Besides mono-ubiquitination of H2A at K119 in Polycomb repression, H2A is also ubiquitinated at the K13/15 site by RNF168 in the early stages of the DNA double-strand break response^34^,^35^. Since K119 and K13/15 reside at distinct locations in the nucleosome (Supplementary Fig. 1e) we asked whether BAP1 could also deubiquitinate K13/15. We tested this by ubiquitinating oligonucleosomal H2A with full-length RNF168 (Supplementary Fig. 1f) before providing the resulting conjugate as a substrate to the BAP1/ASXL1^DEU^ complex. While the activity was low on the K13/15 site, the complex robustly deubiquitinated the Polycomb site indicating a strong preference for this K119 site over the K13/15 site (Fig. 1f).

An interesting question could be whether the specificity is due to the free DNA in the linker of these oligonucleosomes. However, when we analysed specificity on NCPs that have little excess linker DNA, the specificity is retained, indicating that linker DNA does not significantly contribute to specificity (Supplementary Fig. 1g).

Since the N-terminal tail of H2A, that contains K13/15 and the C-terminal tail that contains K119, differ in sequence, the low activity on the K13/15 site could simply reflect a structural incompatibility between the tail and the active site of BAP1. To investigate whether this is the case, we incubated BAP1 and the BAP1/ASXL1^DEU^ complex with N and C-terminal peptides derived from H2A, ubiquitinated on either the K13 or K119, and measured hydrolysis. Peptides were obtained by solid-phase peptide synthesis followed by chemical ubiquitination using thiolysine-mediated ubiquitin conjugation^36^. In both cases, either BAP1 alone or in complex with ASXL1^DEU^ could deubiquitinate the substrates. Moreover the enzyme was more efficient in hydrolysing the K13 site on this peptide (Fig. 1g). These data show that the specificity of the BAP1/ASXL1 complex for the Polycomb site (K119) is not determined by the amino acids surrounding the scissile isopeptide bond, but at regions outside the ubiquitinated tail such as the nucleosome core.

### BAP1 binds ASXL1^DEU^ similar to UCH-L5/RPN13^DEU^

**Figure 2:**
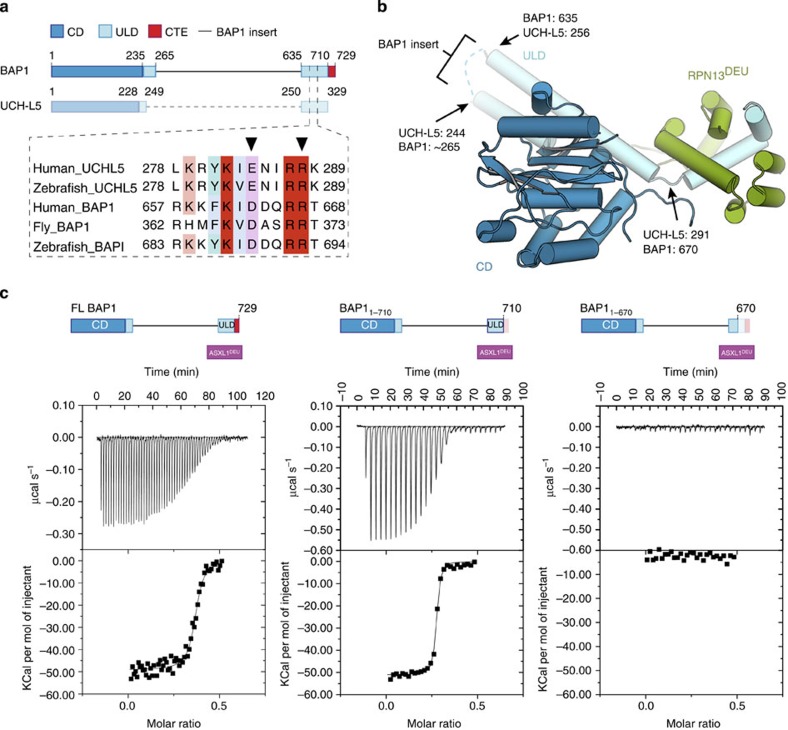
The BAP1 ULD binds ASXL1^DEU^. (**a**) BAP1 and UCH-L5 are highly similar in the catalytic domain and ULD. UCH-L5 does not contain the insert that BAP1 has as indicated by a dashed line. The ULD anchor is highly conserved in a multiple sequence alignment in both BAP1 and UCH-L5 (inset, ULD anchor residues indicated with triangle) (**b**) Cartoon representation of the UCH-L5/RPN13^DEU^ structure(PDB: 4uem ref. 25, UCH-L5 CD in blue, UCH-L5 ULD in light blue, RPN13^DEU^ in green) with labels indicating equivalent BAP1 positions. The predicted location of the BAP1 insert is highlighted (**c**) Isothermal titration calorimetry (ITC) assays. By titrating ASXL1^DEU^ from the syringe to either full-length BAP1 (5 μl injections) or BAP1_1–710_ or BAP1_1–670_ in the cell (both 10 μl injections), we show that ASXL1^DEU^ binds the C-terminal ULD of BAP1 but that the BAP1 CTE is not required for ASXL1^DEU^ binding.

The BAP1/ASXL1 complex has two closely related counterparts, the UCH-L5/RPN13 and UCH-L5/INO80G that are present in the proteasome and in INO80 chromatin remodelling complexes respectively. In these two complexes a DEUBAD domain is present in the regulatory proteins, RPN13 and INO80G, that binds to the C-terminal ULD domain of the UCH-L5. RPN13^DEU^ binding activates UCH-L5 via a combination of mild allosteric effects that increase UCH-L5's affinity for substrates^25^,^26^. First RPN13 positions the active site cross-over loop (CL) in an active conformation. The CL is a flexible loop close to the active site in UCH enzymes that limits enzymatic activity. RPN13^DEU^ binding furthermore precisely anchors the ULD domain to the catalytic domain of UCH-L5 using a highly conserved interface that is also present in BAP1 (Fig. 2a, inset). Since the ULD domain is close to the ubiquitin-binding site, this positioning of the ‘ULD anchor' locks the ULD domain in a conformation that allows ubiquitin binding. This positioning also creates a small interface between ubiquitin and RPN13^DEU^ itself that gives an additional effect on UCH-L5 activation.

The sequence and functional similarity between the BAP1/ASXL1^DEU^ and UCH-L5/RPN13^DEU^ complexes prompted us to examine whether the BAP1/ASXL1 complex functions in a similar fashion (Fig. 2a). We first characterized complex formation by deleting the predicted ASXL1^DEU^-binding site on BAP1, based on the crystal structure of the UCH-L5/RPN13^DEU^ complex (Fig. 2b). As expected, a BAP1 construct lacking the predicted binding site, BAP1_1–670_, did not interact with ASXL1^DEU^ in isothermal titration calorimetry binding assays (ITC; Fig. 2c) and also not in gel filtration (Supplementary Fig. 1h). In contrast, WT BAP1 bound ASXL1^DEU^ with a *K*_*D*_ of 18 nM (Fig. 2c and Supplementary Table 2). These results indicate that BAP1 and UCH-L5 use the same region in the ULD to bind their activator. The ULD of BAP1 is largely similar to UCH-L5 except that it contains an ∼20 aa CTE (Fig. 2a). The CTE is not important for ASXL1^DEU^ binding though, since both full-length BAP1 and BAP1_1–710_ (construct lacking the CTE) could bind ASXL1^DEU^ with similar affinity (Fig. 2c and Supplementary Table 2).

Even though BAP1 and UCH-L5 use the same region to dock their activator, the ITC analysis indicated a notable difference between these complexes. While the UCH-L5/RPN13 complex is equimolar at 1:1, the ligand number in our ITC analysis (between 0.25 and 0.5) suggests that the BAP1/ASXL1^DEU^ complex is asymmetric and consists of multiple BAP1 molecules bound to one ASXL1^DEU^ molecule. To further investigate this, we used size exclusion chromatography (SEC) coupled to multi-angle laser light scattering (SEC-MALLS) experiments, to establish that BAP1 in isolation formed dimers and higher order oligomers (Supplementary Fig. 2a). This oligomerization was abolished upon the addition of ASXL1^DEU^ leading to the formation of a stable BAP1/ASXL1^DEU^ complex with an experimentally determined molecular weight of 179 kDa as calculated by the data analysis software (Supplementary Fig. 2b). This is close to the theoretical 178 kDa molecular weight for a complex consisting of two BAP1 molecules and one ASXL1^DEU^ molecule and far from the MW of a 3:1 or 1:1 complex (260 kDa and 97 kDa respectively) and thus raise the possibility for a 2:1 stoichiometry. ASXL1^DEU^ itself was monomeric in SEC-MALLS (Supplementary Fig. 2c). Collectively, these results are in agreement with a previous quantitative mass spectrometry study that also suggested a 2:1 stoichiometry for the BAP1/ASXL1 complex^15^.

### ASXL1^DEU^ increases BAP1's affinity for ubiquitin

**Figure 3:**
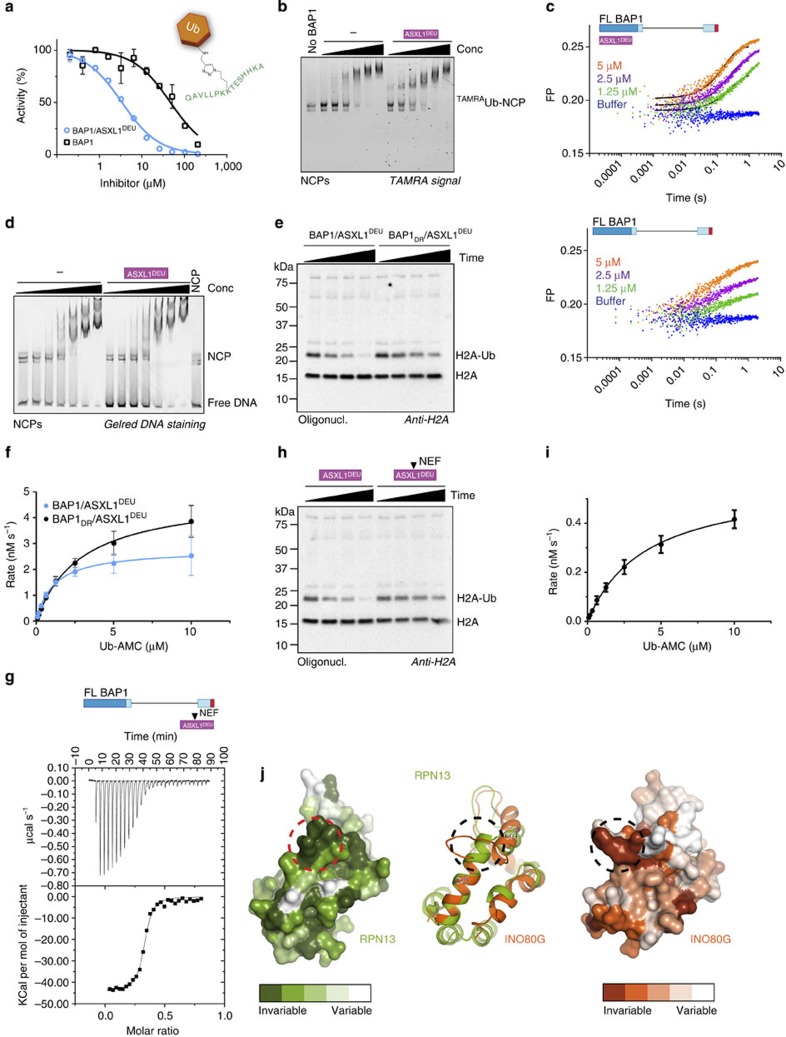
ASXL1^DEU^ activates BAP1 by increasing its affinity for ubiquitin. (**a**) Ub-AMC hydrolysis assay of BAP1 and BAP1/ASXL1^DEU^ in the presence of an H2A peptide that is conjugated to ubiquitin at K119 in a non-hydrolyzable fashion. This conjugate mimics substrates and therefore functions as an inhibitor for Ub-AMC hydrolysis. BAP1 is inhibited more readily by the non-hydrolyzable ubiquitin conjugate in the presence of ASXL1^DEU^. Inset shows the schematic of the non-hydrolyzable H2A ubiquitin conjugate. Error bars, s.d. (*n*=2 independent experiments). (**b**) Native gel shift assay of NCPs mono-ubiquitinated with ^TAMRA^ubiquitin and BAP1 or the BAP1/ASXL1^DEU^ complex. In the presence of ASXL1^DEU^, BAP1 has a higher affinity for mono-ubiquitinated NCPs than BAP1 alone (twofold dilutions starting from 15 μM). Band visualized using the TAMRA signal (**c**) Using stopped-flow fluorescence polarization binding assays we confirmed that the BAP1/ASXL1^DEU^ complex (top) binds better to K119 mono-ubiquitinated NCPs than BAP1 alone (bottom). (**d**) BAP1 and BAP1/ASXL1^DEU^ bind unmodified NCPs with similar affinities in band-shift assays (twofold dilutions starting from 15 μM). Bands visualized using DNA staining by GelRed. (**e**) Anti-H2A western blot showing that the BAP1 ULD anchor mutant (D663A/R667A, DR) complex is less active in deubiquitinating oligonucleosomal H2A than the WT complex (time points: 0, 1, 5, 25 min) (**f**) In Ub-AMC hydrolysis assays, BAP1_DR_/ASXL1^DEU^ complex has a threefold weaker K_M_ than the WT complex. Error bars, s.d. (*n*=3 independent experiments). (**g**) ASXL1_NEF_^DEU^ (N310A/E311K/F312A, NEF) binds BAP1 with similar affinity as WT ASXL1^DEU^ in ITC. (**h** and **i**) ASXL1_NEF_^DEU^ cannot activate BAP1 to the same extent as WT ASXL1^DEU^ using K119 mono-ubiquitinated oligonucleosomal H2A as visualized by western blot (time points: 0, 1, 5, 25 min) or Ub-AMC as a substrate. Error bars, s.d. (*n*=3 independent experiments). (**j**) Surface representation of DEUBAD domains of RPN13 (left) and INO80G (right) indicate high conservation of a region (dotted circle) between helix 4 and 5 (middle: RPN13 green, INO80G orange). Surface conservation was calculated using ConSurf^52^.

ASXL1^DEU^ stimulates BAP1 by lowering the *K*_*M*_ on Ub-AMC (Supplementary Table 1). Therefore we tested whether ASXL1^DEU^ can activate BAP1 by increasing its affinity for ubiquitin. To this end, we analysed the rate of Ub-AMC hydrolysis by BAP1 or the BAP1/ASXL1^DEU^ complex, in the presence of increasing concentrations of a novel non-hydrolyzable H2A-peptide ubiquitin conjugate. The conjugate was obtained using an azido-alkyne click reaction between Ub-75-propargyl (Ub-75-Prg) and azido ornithine followed by thorough purification to remove any traces of Ub-75-Prg, which is a covalent DUB inhibitor^37^. The resulting conjugate mimics substrates^38^ (Fig. 3a) and will therefore compete with Ub-AMC for binding to BAP1 leading to inhibition of Ub-AMC hydrolysis. While the conjugate inhibited BAP1 with an IC_50_ of ∼45 μM, the IC_50_ of the BAP1/ASXL1^DEU^ complex was >10-fold lower indicating that ASXL1^DEU^ induces a higher affinity of BAP1 to the conjugate (Fig. 3a and Supplementary Table 3). The corresponding H2A peptide without a conjugated ubiquitin molecule did not inhibit Ub-AMC hydrolysis at all, and ubiquitin alone had only a minor inhibitory effect on the rate of the BAP1/ASXL1^DEU^ complex (Supplementary Fig. 3a,b). The fact that an ubiquitin conjugate can inhibit BAP1 to a greater extent than ubiquitin alone, illustrates how this DUB prefers binding its target (ubiquitin conjugate) over its product (ubiquitin alone).

To validate this result in a more relevant setting, we performed band-shift assays with NCPs modified with N-terminally TAMRA-labeled ubiquitin (^TAMRA^Ub-NCP) at K119 using the E3 ligase RING1B/BMI1 (Supplementary Fig. 3c). In these assays the BAP1/ASXL1^DEU^ complex shifted ^TAMRA^Ub-NCP more readily compared with BAP1 alone as indicated by the high molecular weight bands appearing already at low concentrations (Fig. 3b). This effect is mainly mediated through BAP1 since ASXL1^DEU^ alone did not bind ^TAMRA^Ub-NCP (Supplementary Fig. 3d). We could confirm our result in a stopped-flow fluorescence polarization binding assay where the BAP1/ASXL1^DEU^ complex could bind to ^TAMRA^Ub-NCPs (*K*_*D*_=4 μM), better than BAP1 alone, which bound so poorly that we could not quantify the affinity constant (Fig. 3c and Supplementary Fig. 3e). Surprisingly, when we tested binding to unmodified NCPs, BAP1 and the complex could shift the NCPs equally well (Fig. 3d). Thus ASXL1^DEU^ specifically promotes the binding of the conjugated ubiquitin and not the nucleosome by itself.

As the region of BAP1 equivalent to the ULD anchor in UCH-L5 (see earlier) is highly conserved (Fig. 2a) we assessed whether it contributes to BAP1 activation. To test this we compared the activity of the ULD anchor mutant of BAP1 (D663A/R667A referred to as DR) bound to ASXL1^DEU^ to the WT complex on two substrates, K119 mono-ubiquitinated oligonucleosomal H2A and Ub-AMC. The ULD anchor mutant complex BAP1_DR_/ASXL1^DEU^ was indeed slightly impaired in H2A deubiquitination and had a weaker *K*_*M*_ than the WT complex in Ub-AMC hydrolysis (Fig. 3e,f and Supplementary Table 1). Therefore we tested whether this *K*_*M*_ effect was due to impaired binding of substrates. For this we used the non-hydrolyzable H2A-peptide ubiquitin conjugate (Fig. 3a) as an inhibitor for Ub-AMC hydrolysis. We found that the mutant complex had a weaker IC_50_ for the conjugate than the WT complex in Ub-AMC assays (Supplementary Fig. 3f and Supplementary Table 3). This suggests that like the UCH-L5/RPN13^DEU^ complex, ASXL1^DEU^ stabilizes the BAP1 ULD domain by anchoring it to the CD allowing more efficient substrate binding.

We next focused our attention on ASXL1^DEU^. After analysis of a multiple sequence alignment of the ASX family we noticed a highly conserved ‘NEF' region that suggests it is important for ASXL1^DEU^ function (Supplementary Fig. 3g). To test this hypothesis we generated a triple mutant containing N310A, E311K and F312A (referred to as ASXL1_NEF_^DEU^). We first validated that ASXL1_NEF_^DEU^ was not impaired in BAP1 binding using ITC (*K*_*D*_=45 nM; Fig. 3g and Supplementary Table 2). Then we tested activity against mono-ubiquitinated oligonucleosomal H2A and found that this mutant was impaired in activating BAP1 compared with WT ASXL1^DEU^ (Fig. 3h). We quantified the activation effect using Ub-AMC as a substrate and noted a threefold weaker *K*_*M*_ but surprisingly also a threefold lower *k*_cat_ compared with WT (Fig. 3i and Supplementary Table 1) conforming that this region of the protein affects BAP1 activation.

To rationalize the effect of the ASXL1^DEU^ ‘NEF' mutant, we examined the crystal structure of the UCH-L5/RPN13^DEU^ complex bound to ubiquitin^25^,^26^. In the structure, the RPN13 region equivalent to the ‘NEF' region stabilizes ubiquitin (Supplementary Fig. 3h) and thereby contributes to activation of UCH-L5. Thus it seems likely that ASXL1^DEU^ also uses this ubiquitin interface to stabilize ubiquitin binding of BAP1 and stimulate activity.

This region in DEUBAD domains in general seems to be important for their function. Analysis of the surface conservation in this region of the DEUBAD domains of UCH-L5 activator RPN13 or inhibitor INO80G, reveals that it is highly conserved within the RPN13 and INO80G proteins (Fig. 3j), but not between these two proteins. In contrast to RPN13^DEU^, which facilitates ubiquitin binding, residues in this conserved region in INO80G form the FRF hairpin that is critical to block ubiquitin binding^25^,^26^. The fact that in all DEUBAD domains, this region is highly conserved and of functional importance, points out that it is a critical site for BAP1/UCH-L5 regulation.

In summary, we have shown that ASXL1^DEU^ activates BAP1 by increasing the affinity for ubiquitin through a combination of mild effects including stabilization of the BAP1 ULD anchor. On the ASXL1^DEU^ side, the highly conserved ‘NEF' region contributes to the activation likely by stabilizing ubiquitin as in the UCH-L5/RPN13^DEU^ complex.

### The BAP1 C-terminus is required for H2A deubiquitination

**Figure 4:**
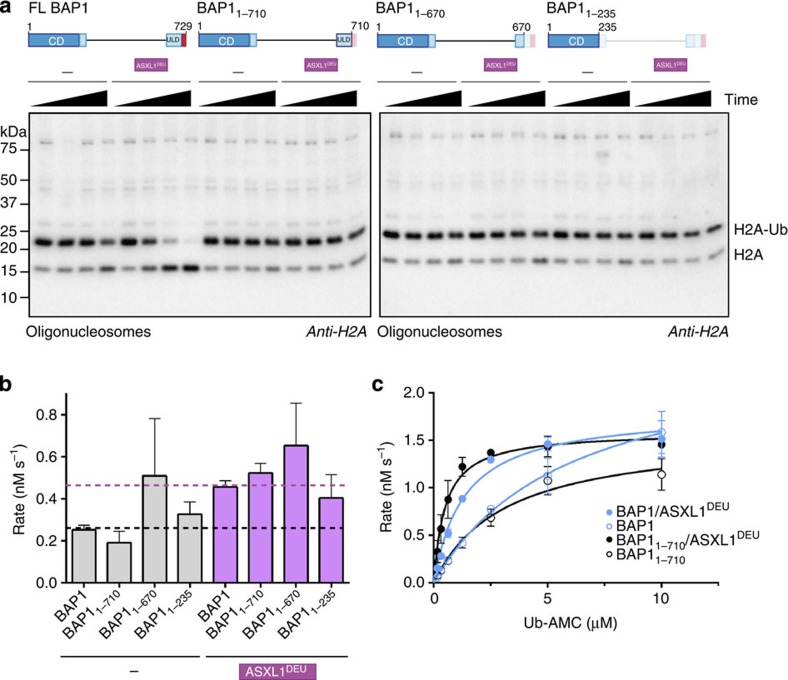
The BAP1 C-terminal extension is required for H2A deubiquitination. (**a**) As shown in an anti-H2A western blot, only WT BAP1 can be activated by ASXL1^DEU^ to cleave ubiquitin from H2A K119 in oligonucleosomes (time points: 0, 1, 5, 25 min). (**b**) All BAP1 variants are active on Ub-AMC. Purple bars represent rates in presence of ASXL1^DEU^. Dotted black and purple lines highlight the rate of WT BAP1 or BAP1/ASXL1^DEU^ respectively. Error bars, s.d. (*n*=3 independent experiments). (**c**): Michaelis–Menten enzyme kinetics showing that like WT BAP1 (blue, curves from Fig. 1b), BAP1_1–710_ (black) can be fully activated by ASXL1^DEU^ (reactions with ASXL1^DEU^ in filled circles) on the minimal substrate Ub-AMC. Error bars, s.d. (*n*=3 independent experiments).

BAP1 is frequently mutated in various tumor types. A subset of mutations in BAP1 result in frame-shifts that lead to premature stop-codons. In some cases, the catalytic domain is still intact suggesting that regions outside this domain are important for proper BAP1 functioning. Therefore we assessed the effect of H2A deubiquitination activity of BAP1 variants truncated at different points: BAP1_1–235_ consisting of only the catalytic domain, BAP1_1–670_, which has a disrupted ULD domain and BAP1_1–710_ that lacks the CTE of BAP1. While the WT BAP1/ASXL1^DEU^ complex was active in H2A deubiquitination, BAP1_1–235_ and BAP1_1–670_ had low activity towards H2A irrespective of the presence of ASXL1^DEU^ (Fig. 4a). This is in accordance with the binding data (Fig. 2c) that indicate that these constructs lack the ASXL1^DEU^ docking site. Surprisingly, BAP1_1–710_ that only lacks the CTE compared with WT and still binds ASXL1^DEU^ similar to WT, had a low activity despite the presence of ASXL1^DEU^ (Fig. 4a and Supplementary Fig. 4a). These results suggested that the BAP1 CTE is important for activity.

To exclude that the intrinsic catalytic activity was affected by the truncation mutants we tested their activity on the minimal substrate Ub-AMC. In contrast to the nucleosomal substrate, we found that all BAP1 variants were active on Ub-AMC, with BAP1_1–235_ and BAP1_1–670_ being even slightly more active than WT demonstrating that the intrinsic activity was not compromised (Fig. 4b). On this model substrate, WT BAP1 and BAP1_1–710_ were equally active in absence of ASXL1^DEU^, and could be activated to the same extent in presence of ASXL1^DEU^ (Fig. 4b,c and Supplementary Table 1). Apparently the CTE does not affect ASXL-dependent activity, but instead it has a substrate-specific role. The tail does not interfere with the intrinsic ability of BAP1 to be activated on the Ub-AMC substrate but is important for activity on the nucleosomal H2A substrate.

### The BAP1 C-terminus auto-recruits BAP1 to nucleosomes

**Figure 5:**
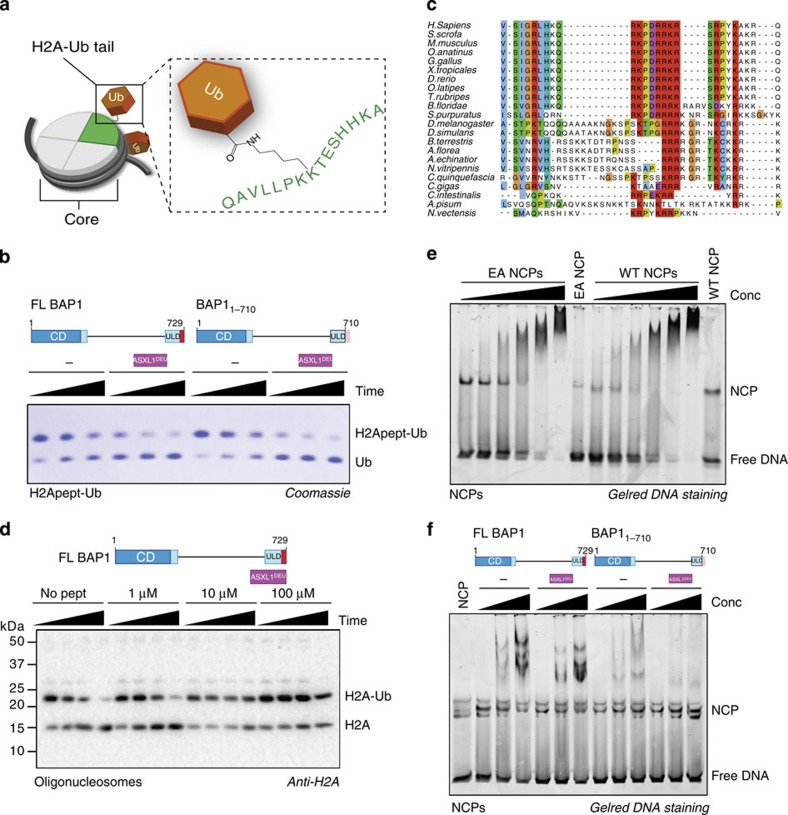
The BAP1 C-terminal extension auto-recruits BAP1 to nucleosomes. (**a**) The nucleosomal H2A substrate can be decomposed into two functional parts: (1) the core consisting of the histone octamers wrapped in DNA and (2) the ubiquitinated H2A tail protruding from the core. (**b**) Coomassie gel of DUB assay using a minimal K119 H2A-ub peptide conjugate substrate. While the BAP1_1–710_/ASXL1^DEU^ complex is inactive on oligonucleosomal H2A, it is as active as the WT complex on the minimal H2A substrate that represents the ubiquitinated H2A tail in isolation (time points: 5, 15, 30 min). (**c**) Multiple sequence alignment of the C-terminus of BAP1 shows that this tail is highly positively charged. (**d**) Anti-H2A blot of a oligonucleosomal H2A K119 deubiquitination assay. Titrating in a synthetic peptide of the BAP1 C-terminus inhibits activity of BAP1/ASXL1^DEU^ (time points: 0, 1, 5, 25 min). (**e**) BAP1 does not require the H2A/H2B acidic patch to bind NCPs, since it shifts WT NCPs as efficient as NCPs where the acidic patch is mutated (EA-NCPs) in band-shift assays (twofold dilutions starting from 15 μM). Shift visualized by DNA staining using GelRed. (**f**) BAP1 that lacks its C-terminal extension (BAP1_1–710_) is impaired in binding NCPs in band-shift assays compared with WT (1, 5, 10 μM of BAP1 variant).

After establishing that the CTE is important on BAP1's natural substrate H2A we sought to determine the basis of this requirement. The nucleosomal substrate has two functional parts; the globular core containing the DNA wrapped around the histone octamer, and the ubiquitinated H2A tail protruding from this core (Fig. 5a). We interrogated the role of the BAP1 CTE by decomposing the nucleosomal substrate and only using a synthetic ubiquitinated H2A tail at K119 as a substrate. On this reduced substrate, both WT and BAP1_1–710_/ASXL1^DEU^ complexes were equally active (Fig. 5b), indicating that the BAP1 CTE is not important at this region of the substrate.

To study how the CTE confers activity on the nucleosomal substrate core (Fig. 5a) we studied a multiple sequence alignment of this region. It contains a nuclear localization sequence^39^, is conserved across species and highly cationic (Fig. 5c). These conserved positive charges may participate in electrostatic interactions with regions of the nucleosome core that tether BAP1 to the substrate. If that was true, co-incubation of BAP1 with a peptide derived from the CTE would compete with the BAP1/ASXL1^DEU^ complex for nucleosome binding leading to decreased DUB activity. We tested this hypothesis, by incubating WT BAP1/ASXL1^DEU^ and BAP1_1–710_/ASXL1^DEU^ with increasing concentrations of a synthetic peptide of the CTE. In absence of peptide, the WT BAP1/ASXL1^DEU^ complex, deubiquitinated H2A robustly as observed before (Fig. 5d). But indeed, in the presence of increasing concentrations of the peptide DUB activity was inhibited for the WT complex (Fig. 5d). The synthetic peptide did not inhibit the intrinsic DUB activity on Ub-AMC demonstrating that the observed effect is specific for nucleosomes (Supplementary Fig. 4b). Interestingly, a scrambled peptide of the CTE also inhibited H2A deubiquitination, suggesting that not the actual sequence but most likely the charge composition is important for the inhibition (Supplementary Fig. 4c).

We then asked if charges on the nucleosome were required for the interaction. An increasing number of chromatin associated proteins are reported to require the acidic patch on nucleosomes for binding or proper functioning^40^,^41^,^42^. This acidic patch is formed at the interface between the H2A/H2B dimer and is prominently solvent exposed. Since the BAP1 CTE is positively charged at physiological pH, we wondered whether it uses the acidic patch to electrostatically recruit itself to nucleosomes in band-shift assays. We found, however, that BAP1 does not require the acidic patch for binding since it shifted WT NCPs and the acidic patch mutant of the NCPs (NCP-EA) in a similar fashion (Fig. 5e). This indicates that BAP1 binds to nucleosomes in a way that is distinct from other nucleosome-binding proteins.

One possibility is that BAP1 associates with nucleosomes by binding DNA. We tested for DNA binding in a competition assay where we measured BAP1/ASXL1^DEU^ activity on H2A K119 ubiquitinated oligonucleosomes in the presence of increasing concentrations of free 146 alpha satellite DNA. No inhibition of DUB activity was observed in the presence of 100 nM or 1 μM DNA while reactions with 10 μM failed to transfer in western blotting (Supplementary Fig. 4d). To still test the effect of 10 μM DNA on the DUB activity we ubiquitinated oligonucleosomes with ^TAMRA^ubiquitin allowing us to circumvent western blotting and instead directly visualize the substrate using the TAMRA signal. When incubating BAP1/ASXL1^DEU^ with the fluorescent substrate, robust deubiquitination occurred even in the presence of 10 μM DNA (Supplementary Fig. 4e). Since DNA cannot compete with the reaction, we conclude that recruitment of BAP1 to nucleosomes does not depend on DNA binding.

Finally we tested if the CTE was important for BAP1 recruitment to the nucleosome in a direct-binding assay. In band-shift assays, BAP1/ASXL1^DEU^ could shift the NCP whereas BAP1_1–710_/ASXL1^DEU^ complex was less capable of shifting the NCP indicating again that the C-terminus is important for binding to nucleosomes (Fig. 5f). In short, these results are consistent with a model where the BAP1/ASXL1^DEU^ complex has two main binding components. The BAP1 CTE is required for binding to the NCP core, and the presence of ASXL1^DEU^ increases BAP1's affinity for the ubiquitin moiety at the protruding H2A tail.

## Discussion

BAP1 is a critical tumor suppressor that is frequently mutated in human cancer. In this paper we describe that similar to the homologous UCH-L5/RPN13^DEU^ complex, the DEUBAD domains of ASXL1, ASXL2 and ASXL3 can activate BAP1 by increasing BAP1's affinity specifically for the ubiquitin in the substrate, through a combination of mild effects. We observed that activation by ASXL proteins is not sufficient for deubiquitination of BAP1's natural substrate H2A. This also requires the BAP1 CTE that binds to nucleosomes.

**Figure 6:**
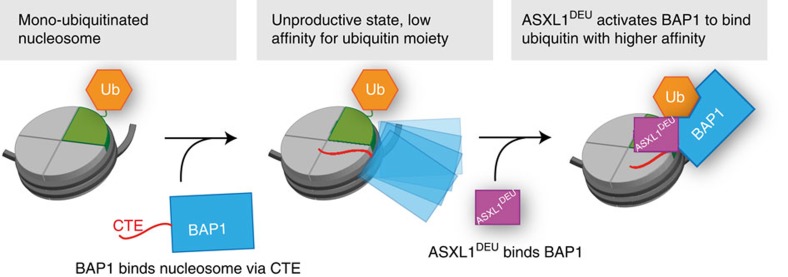
Model for H2A deubiquitination by the BAP1/ASXL1 complex. BAP1 (blue) binds nucleosomes mono-ubiquitinated at K119 using its C-terminal extension (red). This complex has low DUB activity owing to BAP1's low affinity for ubiquitin on H2A (orange). Binding of the DEUBAD domain of ASXL1 (purple) allosterically activates BAP1 by increasing its affinity for the ubiquitin moiety on H2A, driving the DUB reaction.

We propose a model where H2A deubiquitination by the BAP1/ASXL1^DEU^ complex consists of two key processes. The BAP1 CTE tethers BAP1 to the nucleosome core. This initial encounter-like complex is however unproductive owing to BAP1's low affinity for the mono-ubiquitinated H2A tail in absence of an activator. In a second key process ASXL1^DEU^ can convert this complex into a productive one by increasing BAP1's affinity for ubiquitin on H2A (Fig. 6).

The mechanism by which ASXL1^DEU^ increases BAP1's affinity for ubiquitin is related to that of the UCH-L5/RPN13^DEU^ complex of which crystal structures were reported recently^25^,^26^. RPN13^DEU^ activates UCH-L5 by decreasing the mobility of the ULD domain, a domain close to the ubiquitin-binding site. By fixating it to the UCH-L5 CD via the ULD anchor, RPN13^DEU^ exposes the ubiquitin docking site on UCH-L5 thereby increasing the affinity for ubiquitin. RPN13^DEU^ also mildly contributes to UCH-L5 activation by directly contacting ubiquitin, and by positioning the active site CL. The CL is a flexible loop close to the active site in UCH enzymes that limits enzymatic activity. Individually these effects are mild but combined they provide substantial regulation.

For BAP1 we observe similar effects, in agreement with a report that appeared during revision of our manuscript^43^. The ULD anchor has a mild effect on affinity, and the region on ASXL1^DEU^ that is equivalent to the region of RPN13^DEU^ that contacts ubiquitin also affects BAP1 activity. The effect of the latter is, however, stronger in ASXL1^DEU^ than in RPN13^DEU^. This highlights that although the regulatory regions are similar in the UCH-L5/RPN13^DEU^ or BAP1/ASXL1^DEU^ complex, their relative individual contribution to activation may be different. The lack of conservation between the UCH-L5 and BAP1 CLs made it difficult to assess whether ASXL1^DEU^ also positions the BAP1 CL towards an active conformation. Therefore we omitted this aspect from our analysis but we do not exclude that this may occur.

Our data suggest that the BAP1 CTE is an important component for nucleosome binding. This binding already occurs in the absence of the ubiquitin mark on H2A. Even though BAP1 is recruited to nucleosomes by various proteins in cells, it is possible that BAP1's relative orientation towards H2A-Ub in these complexes is suboptimal for substrate binding and catalysis. This could be the reason why the BAP1 CTE is required. In addition to the function ascribed to the BAP1 CTE in this study, others have shown that it also hosts a nuclear localization signal that can be ubiquitinated by UBE2O to mislocalize BAP1 to the cytoplasm^39^,^44^ underscoring the importance of this small region of BAP1. The multiple functions of the CTE also make analysis of its importance in cells difficult.

While ASXL1 and ASXL2 were known to interact with BAP1 from proteomics studies^8^,^33^, ASXL3 was not detected as a BAP1-binding partner, possibly because of the low levels of BAP1/ASXL3 complexes in cells. Our *in vitro* analysis indicates that also ASXL3 can bind and activate BAP1, which is not surprising in the light of the high sequence similarity between the DEUBAD domains in the ASXL family. It suggests that there may be a functional association between BAP1 and ASXL3 in cells and further cell based work is required to shed more light on this issue.

We found that in a nucleosomal context, the BAP1/ASXL^DEU^ complex is specific for H2A mono-ubiquitinated at the Polycomb site K119 and not on the DNA-damage site K13/15. BAP1 has been suggested to have a role in DNA repair but its exact role is unclear. Our results indicate that it is unlikely that the BAP1/ASXL1 complex regulates the DNA-damage response by deubiquitinating H2A at K13/15. The specificity for the Polycomb site may be governed by interfaces of the complex with the nucleosome. Therefore we also assessed whether BAP1 can bind the acidic patch on the nucleosome and found that the complex does not rely on this site for binding unlike other nucleosome-binding proteins.

An unexpected finding was the stoichiometry of the BAP1/ASXL1 complex, that was not 1:1 in ITC analysis. Our SEC-MALLS analysis, furthermore, suggests that BAP1 forms higher order oligomers that are disrupted by ASXL1^DEU^ binding to favour, what is most likely a complex consisting of two BAP1 molecules and one ASXL1^DEU^ molecule. More studies will be needed to investigate this intriguing observation. DUB dimers are not unusual though. The JAMM proteins RPN11 and BRCC36 both require hetero-dimerization with RPN8 and Abro1 or Abraxas, respectively, but in these cases only the first protomer is active. It may also be possible that only one subunit is active in the BAP1/ASXL1 complex. It is conceivable that the BAP1 dimerization is a manifestation of selective pressures on BAP1 to be part of multi-protein complexes where one monomer functions as an assembly platform for other proteins. Future studies will be necessary to further examine the possible functional implications of the BAP1/ASXL1 stoichiometry.

BAP1 associates with many factors to form multi-protein complexes. One of the main outstanding questions is whether multiple mutually exclusive BAP1 complexes exist and whether BAP1 catalytic activity is required in all of these complexes. Perhaps BAP1 can also serve as a scaffold in some of these complexes to recruit other chromatin modifiers. An important step in understanding the carcinogenesis in BAP1 deficiency will therefore rely on a thorough understanding of the different BAP1 complexes and the effects on BAP1 activity. Our comprehensive biochemical analysis of binding interfaces, activation mechanism and recruitment factors of the BAP1/ASXL1 complex provides a framework for future work on the possible role of these larger BAP1 complexes in tumorigenesis.

## Methods

### Plasmids and cloning

Full-length cDNA's for BAP1 (uniprot Q92560), ASXL1 (uniprot Q8IXJ9) and ASXL2 (uniprot Q76L83) were kindly provided by Dr Jürg Müller. cDNA for the ASXL3 DEUBAD domain (uniprot Q9C0F0) was purchased as a codon optimized gBlock (Integrated DNA Technologies). All constructs were cloned into expression vectors of the NKI Ligation Independent Cloning Suite^45^. Full-length BAP1, BAP1_1–710_ and BAP1_1–670_ were cloned into pFastBac-NKI-his-3C-LIC vector; BAP1_1–235_ into pET-NKI-strepII-3C-LIC; ASXL1^DEU^, ASXL2^DEU^, ASXL3^DEU^ and ASXL1_1–390_ into pET-NKI-his-3C-LIC (carbenicilin); and finally ASXL1^HH^ into pGEX-NKI-3C-LIC. All clones were verified by DNA sequencing.

### Protein expression and purification

Full-length BAP1, BAP1_1–710_, BAP1_1–670_, full-length Ring1b/Bmi1 and their variants were expressed as His-tagged fusion constructs in Sf9 cells for 48–72 h at 27 °C, whereas His-ASXL1^DEU^ (aa 238–390), His-ASXL2^DEU^ (aa 261–380), His-ASXL3^DEU^ (aa 244–360), His-ASXL1_1–390_, GST-ASXL1^HH^ (1–94), StepII-BAP1_1–235_ and their variants were expressed in *Escherichia coli*. For bacterial expression, cells were grown to an OD of 0.6 at 37 °C before inducing expression with 0.5 mM IPTG at 25 °C for 4–6 h. All cells were lysed by either bead beating or sonication using lysis buffer (50 mM Tris pH 8.0, 200 mM NaCl, 50 mM Imidazole pH 8.0, 0.5 mM TCEP) supplemented with complete EDTA-free protease inhibitor cocktail (Roche).

For the insect-cell-expressed constructs, cleared lysates were incubated with chelating sepharose beads (GE Healthcare) pre-charged with Ni^2+^. After washing with >15 column volume (CV) lysis buffer and elution with elution buffer (20 mM Tris pH 8.0, 150 mM NaCl, 500 mM Imidazole pH 8.0, 10% Glycerol and 0.5 mM TCEP), the sample was purified in anion exchange chromatography using Poros HQ resin (Applied Biosystems). A linear salt gradient from 0–75% buffer B (20 mM Tris pH 8.0, 1 M NaCl, 5% Glycerol, 0.5 mM TCEP) was used to elute the protein from the column that was previously equilibrated in buffer A (20 mM Tris pH 8.0, 50 mM NaCl, 5% Glycerol, 0.5 mM TCEP). As a final step, the sample was fractionated on a Superose6 (GE Healthcare) SEC column for full-length BAP1 and BAP1_1–710_ or on a Superdex 200 SEC column for BAP1_1–670_ in gel filtration buffer (10 mM HEPES pH 7.5, 150 mM NaCl, 10% Glycerol and 0.5 mM TCEP). Presence of BAP1 was verified through western blotting using a 1:1,000 dilution of anti-BAP1 (abcam, ab167250).

The BAP1 catalytic domain was purified using StrepTactin beads (GE Healthcare). After washing the beads with <10 CV lysis buffer, protein was eluted with Strep elution buffer (20 mM Tris pH 8.0, 150 mM NaCl, 10% Glycerol, 2.5 mM Desthiobiotin and 0.5 mM TCEP). This was followed by a final SEC step using a Superdex 75 (GE Healthcare) equilibrated in gel filtration buffer.

Cleared lysates of ASXL constructs were purified using His-tag affinity chromatography using chelating sepharose beads (GE Healthcare) pre-charged with Ni^2+^. After washing with >10 CV lysis buffer and elution with elution buffer (20 mM Tris pH 8.0, 100 mM NaCl, 500 mM Imidazole pH 8.0, 10% Glycerol and 0.5 mM TCEP), the sample was purified in anion exchange chromatography using Poros HQ resin (Applied Biosystems) for the DEUBAD domains. A linear salt gradient from 0–75% buffer B (20 mM Tris pH 8.0, 1 M NaCl, 5% Glycerol, 0.5 mM TCEP) was used to elute the protein from the column that was previously equilibrated in buffer A (20 mM Tris pH 8.0, 50 mM NaCl, 5% Glycerol, 0.5 mM TCEP). For the ASXL1^HH^ and ASXL1_1–390_ constructs, a cation exchange step using a Poros HS (Applied Biosystems) was introduced instead of the anion exchange step. Here, a linear salt gradient from 0–75% buffer B (20 mM Bis-Tris pH 6.5, 1 M NaCl, 5% Glycerol, 0.5 mM TCEP) was employed to elute the protein from the column that was previously equilibrated in buffer A (20 mM Bis-Tris pH 6.5, 50 mM NaCl, 5% Glycerol, 0.5 mM TCEP). The final step was SEC using a Superdex 75 (GE Healthcare) column equilibrated in gel filtration buffer.

Full-length RING1B/BMI1-his was lysed in 50 mM Tris pH 8.0, 500 mM NaCl, 50 mM Imidazole pH 8.0, 10% Glycerol, 2 μM ZnCl_2_ and 0.5 mM TCEP. The cleared lysate was incubated with chelating sepharose beads (GE Healthcare) pre-charged with Ni^2+^. After washing with >10 CV lysis buffer and elution with elution buffer (50 mM Tris pH 8.0, 500 mM NaCl, 500 mM Imidazole pH 8.0, 10% Glycerol, 2 μM ZnCl_2_ and 0.5 mM TCEP). Hereafter, the sample was fractionated on a Superdex 200 SEC column (GE Healthcare) in 10 mM HEPES pH 7.5, 400 mM NaCl, 10% Glycerol, 2 μM ZnCl_2_ and 0.5 mM TCEP. Full-length RNF168 was purified as described before^34^.

After SEC, all samples were concentrated using AMICON Ultra concentration columns (Millipore) to 2–20 mg ml^−1^ and flash frozen in liquid nitrogen.

### Oligonucleosome purification

HEK293T cells were cultured in DMEM containing 10% foetal calf serum. The cell membranes of ∼300 million HEK293T cells were rendered porous by washing them twice with 5 cell pellet volumes of 10 mM Tris pH 8.0, 1.5 mM MgCl_2_, 10 mM KCl and 0.5 mM DTT (Buffer A)^34^. After the final wash, cells were taken up in two cell pellet volumes of buffer A and lysed, while keeping the nuclei intact, using the loose pestle of a dounce homogenizing device. After lysis the nuclei were pelleted by centrifugation and resuspended in 1.5 ml (1 ml per 200 million nuclei) of buffer M1 (50 mM Tris pH 7.5, 60 mM KCl, 3 mM CaCl_2_, 0.34 M Sucrose) supplemented with complete protease inhibitor tablets (Roche). Next, the chromatin was digested at 37 °C for 10 min with micrococcal nuclease (Worthington) by the addition of 60 U ml^−1^ enzyme (final concentration) yielding a mixture of di, tri and tetranucleosomes. The digestion reaction was stopped by adding 50 mM of EGTA after which the nuclei were disrupted by douncing the mixture on ice × 100 up and down, using the tight pestle of a dounce homogenizer. Next, the sample was rotated end over end in the cold room, after adding 500 mM NaCl (final concentration) to the samples. After centrifugation, the nucleosomes were recovered from the supernatant and dialyzed overnight in the cold room against EQ buffer (20 mM HEPES pH 7.5, 650 mM NaCl, 2 mM EDTA pH 8.0, 1 mM TCEP, 0.5 mM PMSF, 2 mM Benzamidine and 16 mM beta-glycerol-phosphate). As a final purification step, the sample was fractionated on a Superose 6 10/30 (GE Healthcare), concentrated and flash frozen for storage at −80 °C.

### Oligonucleosome DUB assays

Purified oligonucleosomes (2–3 μM as estimated from coomassie gel) were mono-ubiquitinated at Lys119 in a reaction mixture that also contained 500 nM Uba1, 2 μM UbcH5c, 2 μM RING1B/BMI1 and 7.5 μM Ubiquitin or ^TAMRA^Ubiquitin. For ubiquitination of H2A K13/15, 1 μM of full-length RNF168 was used. The reaction took place for 1 h in reaction buffer (25 mM HEPES pH 7.5, 150 mM NaCl, 2 μM ZnCl_2_, 5 mM DTT, 5 mM ATP and 3 mM MgCl_2_) at 30 °C. NCPs were ubiquitinated under the same conditions. Reactions were terminated by depleting ATP using Apyrase (Sigma-Aldrich). The DUB reaction was performed in 10 μl reactions at 30 °C using 50–100 nM BAP1 variant or BAP1/ASXL1^DEU^ variant, and between 1.5 and 2.25 μM of ubiquitinated oligonucleosomes or NCPs reconstituted with 146 alpha satellite DNA. Prior to the reaction the BAP1/ASXL1 complexes were allowed to form on ice for 10–30 min. Reactions were terminated by the addition of SDS-PAGE protein-loading buffer and analysed by western blotting using 1:1,000 dilution of anti-H2A (07–146, Millipore) or using the TAMRA signal of ^TAMRA^Ubiquitin.

### Reconstitution of NCPs

Recombinant NCPs were prepared as described previously^40^,^46^,^47^,^48^. Briefly, Xenopus histones were expressed and purified from *E. coli* and assembled into histone octamers that were purified via SEC. NCPs were formed by incubating the octamer with either the 167 bp Widom 601 or 146 bp alpha satellite strong DNA positioning sequences using a linear salt gradient from 2 M to 150 mM KCl using a peristaltic pump (Watson Marlow pumps). To avoid sample heterogeneity owing to slightly different DNA positioning, the reconstituted NCPs were subjected to a heat shift by incubating the samples at 37 °C for 30 min.

### Ub-AMC enzymatic assays

Enzyme activity was followed as the release of fluorescent AMC from the quenched Ub-AMC substrate, providing a direct readout of DUB activity^32^. Ub-AMC was synthesized as described previously^36^. The purified Ub-AMC was dissolved in pure DMSO. The residual amount of DMSO left in the enzymatic reaction was never higher than 3%. Kinetic parameters were determined using 1 nM of enzyme while varying the substrate concentration (6 times 1:1 dilution starting from the highest concentration of 10 μM) in 30 μl reactions using reaction buffer (25 mM HEPES pH 7.5, 150 mM NaCl, 5 mM DTT and 0.05% Tween-20) at 25 °C. Reactions took place in black 384-well non-binding surface low flange plates (Corning). In the single concentration experiments, 1 nM of enzyme was allowed to react with 1 μM of substrate and activity was quantified by calculating the initial rates. Experiments were performed in a Pherastar (BMG Labtechnologies) plate reader using 350 and 450 nm excitation and emission wavelengths respectively. Measurements were taken every 10 s for 10 min. Fluorescence and velocities were related using an AMC standard curve. The initial rates were plotted against substrate concentration and fitted to the Michealis–Menten model using non-linear regression in Prism 6 (GraphPad software).

For the inhibition assays, increasing concentrations of the non-hydrolyzable H2A ubiquitin conjugates (10 times 1:1 dilution starting from the highest concentration of 200 μM) were incubated with 500 pM enzyme and 2 μM of substrate and for each concentration the initial rates were calculated. The rates were normalized by setting the no-inhibitor control at 100% activity. Inhibition data were fitted to a ‘log inhibitor versus normalized response' model to obtain IC_50_ values in Prism 6 (GraphPad software).

### Synthesis non-hydrolyzable H2A-Ub conjugates

The H2A-derived peptide was obtained through standard linear FMOC-based peptide synthesis on WANG resin^36^. The target lysine in the peptide (K119) was substituted for Azidonorvaline (incorporated as Fmoc-azidonorvaline). Ubiquitin with a C-terminal propargyl (Ub-Prg) was obtained as described previously^37^. The Tris triazole ligand (tris(1-(O-ethylcarboxymethyl)-1H-1,2,3-triazol-4-ylmethyl)amine) used in the click reactions was synthesized as previously described^49^. For the reaction to be successful it is important that the source of Cu(I) is pure and free of oxidized byproduct, Cu(II). CuBr was obtained in 99% purity and was a green/brown powder indicating traces of Cu(II). Cu(II)Br was suspended 10% v/v in high purity (glacial) acetic acid. This suspension was vigorously stirred under nitrogen overnight. After stirring overnight the suspension was filtered and the off-white residue separated from the greenish solution. The solid was washed with ethanol and dried under high vacuum. The obtained off-white powder was stored under inert atmosphere.

Both the functionalized H2A peptide and Ub-Prg dissolved in DMSO at 50 mg ml^−1^ and heated to facilitate solubilization. The peptide was dissolved in DMSO at 50 mg ml^−1^ and heated in case it failed to dissolve. Ub-Prg was dissolved in DMSO at 50 mg ml^−1^ and heated to make a proper solution. To 1 ml of 8 M urea (0.1 M phosphate buffer pH 8), was added 100 μl of Ub-Prg solution and 100 μl of peptide solution. After mixing the peptide and the ubiquitin the click solution was prepared.

A fresh solution of CuBr was prepared (20 mg ml^−1^ in acetonitrile) and a stock solution of ligand (50 mg ml^−1^ in acetonitrile were used. 19.2 μl of CuBr solutions was added to 30 μl of ligand solution. The Cu/ligand solution was then added in five equal steps to the reaction mixture, each addition being vortexed and left for a few minutes before adding another 10 μl of Cu/ligand mix. The progression of the reaction was checked using LC-MS. After completion of the reaction 50 μl of a 0.5 M EDTA pH 7 solution was added to quench the reaction and dissolve remaining Cu(II) species. As a final step, the reaction mixture was applied directly to reversed-phase HPLC for purification. Fractions were analysed for presence of product, pooled and lyophilized.

### H2A-peptide conjugate hydrolysis assay

Native H2A-peptide (KQGGKARAKAKTRSSRA for K13 and QAVLLPKKTESHHKA for K119) ubiquitin conjugates were chemically synthesized as described previously^36^,^50^. 20 μM of conjugate was incubated with 100 nM of enzyme or enzyme complex in reaction buffer (25 mM HEPES pH 7.5, 150 mM NaCl and 5 mM DTT and incubated at 30 °C. Reactions were terminated by the addition of protein-loading buffer and analysed by SDS-PAGE.

### Band-shift assays

Band-shift assays were performed using native gel electrophoresis on 4–12% Pre-Cast Tris-Glycine gels (Life Technologies), pre-run for at least 1 h at 125 V in Novex Tris-Glycine buffer at 4 °C. NCPs or mono-ubiquitinated NCPs (both 100 nM), reconstituted with the Widom 601 167 bp DNA positioning sequence, were incubated with increasing amounts of BAP1 variant or ASXL1^DEU^ or BAP1/ASXL1^DEU^ variant and the gel was run for 90 min at 125 V at 4 °C. BAP1/ASXL1^DEU^ complexes were allowed to form for 10–30 min on ice prior to electrophoresis. Bands were visualized by DNA staining with GelRed or using the TAMRA signal in a ChemiDoc XRS instrument (Biorad).

### Isothermal Titration Calorimetry

ITC experiments were performed in a VP-ITC Microcal calorimeter (Malvern) at 25 °C. All proteins were dialyzed to ITC buffer (25 mM HEPES pH 7.5, 150 mM NaCl, 10% glycerol, 0.5 mM TCEP) prior to the experiment. Using 10 or 5 μl injections, 30 μM ASXL1^DEU^ was titrated into 13 μM of BAP1 variant. Data were fitted with a one-site binding model script in the manufacturer's Origin software.

### Stopped-flow fluorescent polarization binding assays

Pre-steady state binding events between inactive mutants (C91A) of BAP1 or BAP1/ASXL1^DEU^ and NCPs, reconstituted with Widom 601 167 bp DNA and monoubiquitinated with TAMRA-ubiquitin at K119 (^TAMRA^Ub-NCP) by Ring1b/Bmi1, were monitored in stopped-flow fluorescent polarization experiments. The experiments were performed on a stopped-flow system (TgK Scientific model SF-61DX2) equipped with a photomultiplier tube R10699 (Hamamatsu). Monochromatic light at 544 and a 570 nm cutoff filter were used for excitation and emission readout, respectively. The light was polarized using a calcite prism for the incident beam and dichroic sheet polarizers in front of each of two photo-multiplier detectors arranged in a T-configuration.

The experiments were performed in stopped-flow binding buffer (25 mM HEPES pH 7.5, 150 mM NaCl, 0.05% Tween-20 and 0.5 mM TCEP) at 20 °C. For the association 20 nM of ^TAMRA^Ub-NCP (final concentration) and various concentrations of BAP1 or BAP1/ASXL1^DEU^ were injected in equal volumes and rapidly mixed after which FP signal was followed during 10 s. For each concentration three injections were averaged to improve signal. Association binding traces were fitted to a one-phase association model in Prism 6 (GraphPad software) to obtain *k*_obs_. The *k*_obs_ values were plotted against protein concentration and analysed by linear regression to estimate *k*_on_, *k*_off_ and -*K*_*D*_. BAP1 association traces did not come close to saturation and were therefore not fitted.

### Surface conservation DEUBAD domains

Twenty or more sequences across all eukaryotic lineages containing both UCH-L5 and RPN13 or INO80G where aligned using the MAFFT algorithm^51^. Surface conservation calculation were performed using ConSurf^52^, with default settings. PBD codes for the DEUBAD domains are 4uem and 4uf5. The structures of the DEUBAD domains where superimposed using pdbeFOLD^53^.

### Multi-angle laser light scattering

Multi-angle laser light scattering experiments were performed using a miniDawn light scattering detector (Wyatt Technologies) in line with either a Superdex S75 10/30 (ASXL1^DEU^), Superose 6 10/30(BAP1) or Superdex S200 10/30(BAP1/ASXL1^DEU^) SEC column (GE Healthcare). SEC-MALLS runs were performed in 10 mM HEPES pH 7.5, 150 mM NaCl and 0.5 mM TCEP at 4 °C. Molecular weights were calculated by using the refractive index signal with the Astra software (Wyatt Technologies).

## Additional information

**How to cite this article**: Sahtoe, D. D. *et al.* BAP1/ASXL1 recruitment and activation for H2A deubiquitination. *Nat. Commun.* 7:10292 doi: 10.1038/ncomms10292 (2016).

## Supplementary Material

Supplementary Information supplementary Figures 1-4 and Supplementary Tables 1-3
