# Supplementary material for: BAP1/ASXL1 recruitment and activation for H2A deubiquitination
Source: Nat Commun. 2016 Jan 7;7:10292. doi: 10.1038/ncomms10292 (PMC4729829; doi:10.1038/ncomms10292)
Supplement: Supplementary Information — supplementary Figures 1-4 and Supplementary Tables 1-3 [file ncomms10292-s1.pdf]

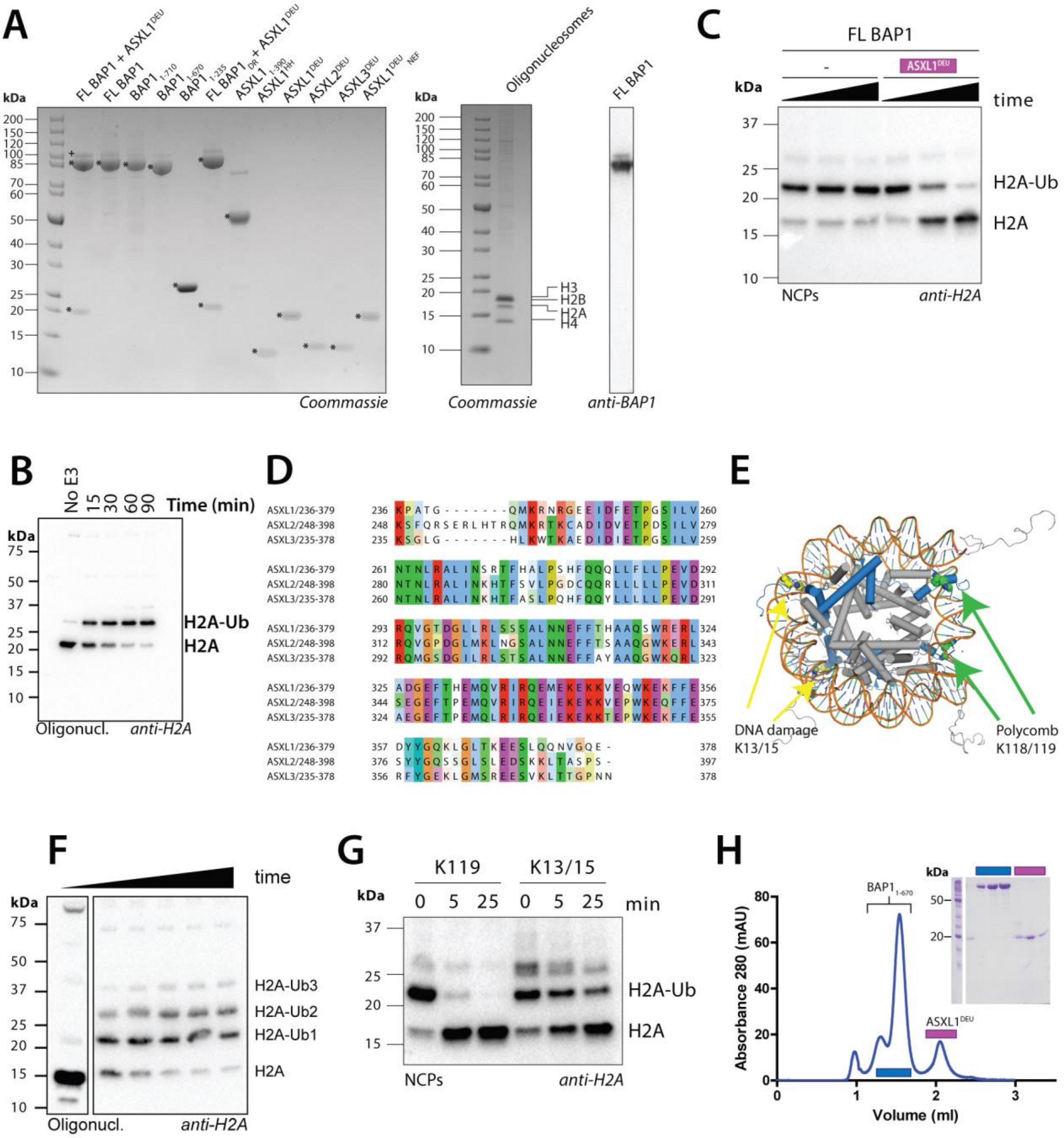

Supplementary Figure 1. ASXL family DEUBAD domains activate BAP1 for specifically H2A K119 deubiquitination. (a) Coommassie gel of preparations used in this study. Asterisks highlight bands of interest. The species indicated by a "+" are BAP1 molecules modified post-translationally in insect cells and not co-purified contaminant proteins as confirmed by anti BAP1 (abcam, ab167250) western blotting (right panel). **(b)** Anti-H2A western blot of the monoubiquitination of oligonucleosomal H2A K119 by full length Ring1b/Bmi1. **(c)** The DEUBAD domain of ASXL1 also activates BAP1 on NCPs as visualized by anti-H2A western blotting. Time points: 0, 1, 5 min. **(d)** Multiple sequence alignment of the DEUBAD domains in human ASXL1, ASXL2 and ASXL3 using the MAFFT algorithm. The sequence identity among the family members is >60%. **(e)** The Polycomb (green spheres) and DNA damage (yellow spheres) ubiquitination sites on H2A (blue) are situated at distinct locations in the nucleosome core particle. **(f)** Anti-H2A blot in which the ubiquitination of lysine 13 or 15 by full length RNF168 is followed (time points: 0, 15, 30, 45, 60, 90 min). Time point 0 was not adjacent to other time points on gel. **(g)** Anti-H2A blot of DUB activity of the BAP1/ASXL1^{DEU} complex on ubiquitinated H2A at K119 and K13/15 in the context of NCPs reconstituted with the 146 bp alpha satellite DNA positioning sequence. Unlike on oligonucleosomes, no linker DNA is present in NCPs and yet the complex retains specificity for the K119 site showing that the specificity is not conferred by the DNA. **(h)** BAP1$_{1\text{-}670}$ and ASXL1^{DEU} do not co-elute when applied to a S200 5/150 size exclusion chromatography column indicating that the BAP1$_{1\text{-}670}$ construct does not bind ASXL1^{DEU} in agreement with the ITC analysis. The inset shows BAP1$_{1\text{-}670}$ fractions (blue bar) and ASXL1^{DEU} fractions (purple bar on SDS-PAGE.

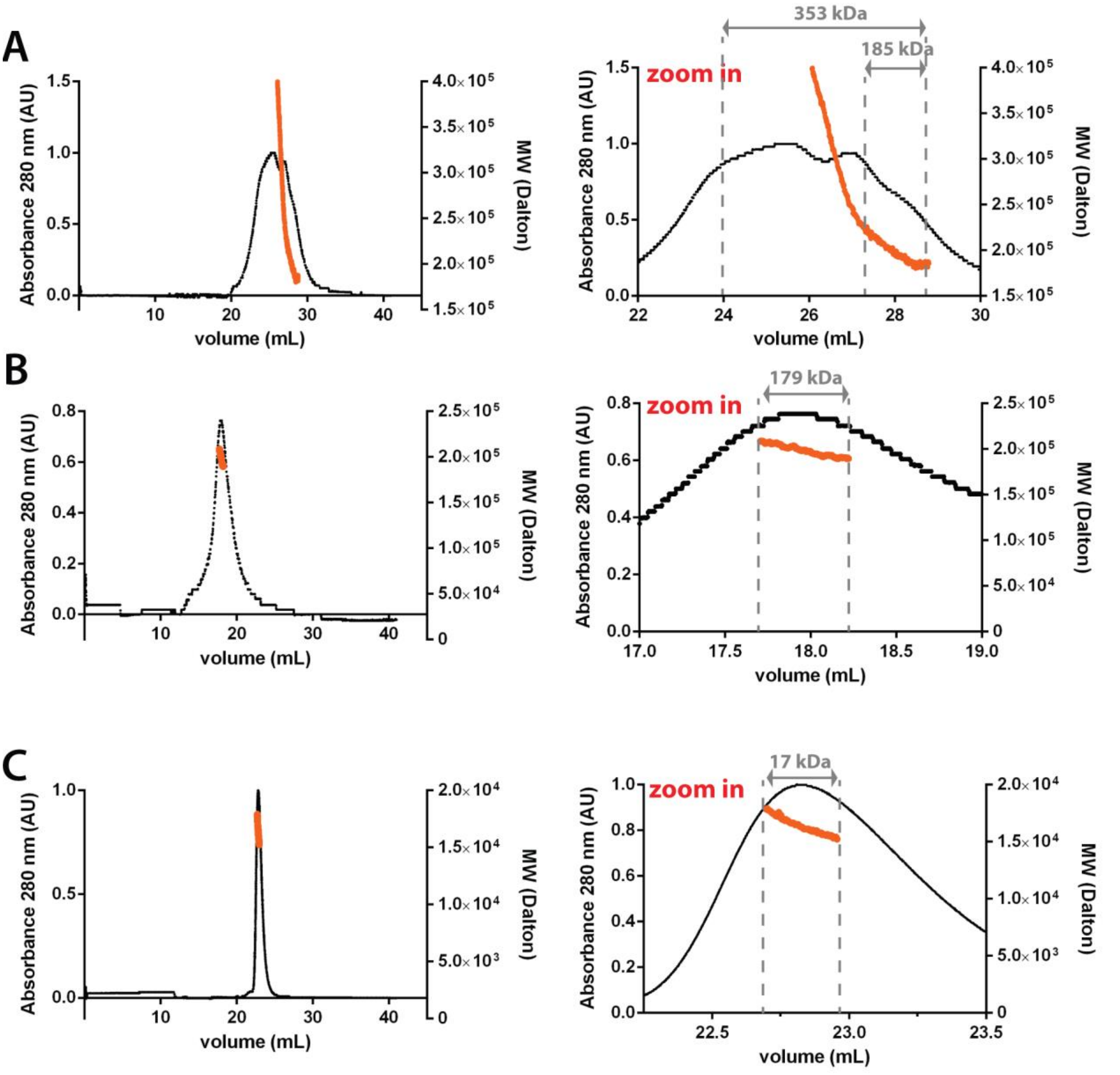

Supplementary Figure 2. Oligomeric state of the BAP1/ASXL1 complex. (a) Left panel: BAP1 superose 6 10/30 SEC-MALLS graph (SEC: black, MALLS: orange). Right panel: Zoom in of the peak. Two regions of the peak were selected for analysis (dotted lines). The right side of the peak where the MALLS signal (orange) flattens was determined to have a 185 kDa molecular weight using the refractive index (RI) signal, consistent with a BAP1 dimer (theoretical MW 160 kDa). The entire peak was calculated to have a 353 kDa molecular weight. This value is likely inaccurate however due to the formation of higher order oligomers. All SEC-MALLS data were analyzed using the Astra software (Wyatt Technology). **(b)** The BAP1/ASXL1^{DEU} complex is 179 kDa as calculated from superdex S200 10/30 SEC-MALLS analysis. Peak subjected to analysis indicated by dotted lines. The result is consistent with the 2:1 (theoretical MW 178 kDa) complex suggested by our ITC analysis and mass spec analysis (Baymaz et al 2014) but far from a 1:1 complex (theoretical MW 100 kDa). **(c)** ASXL1^{DEU} is monomeric (theoretical MW 18 kDa) in superdex S75 10/30 SEC-MALLS.

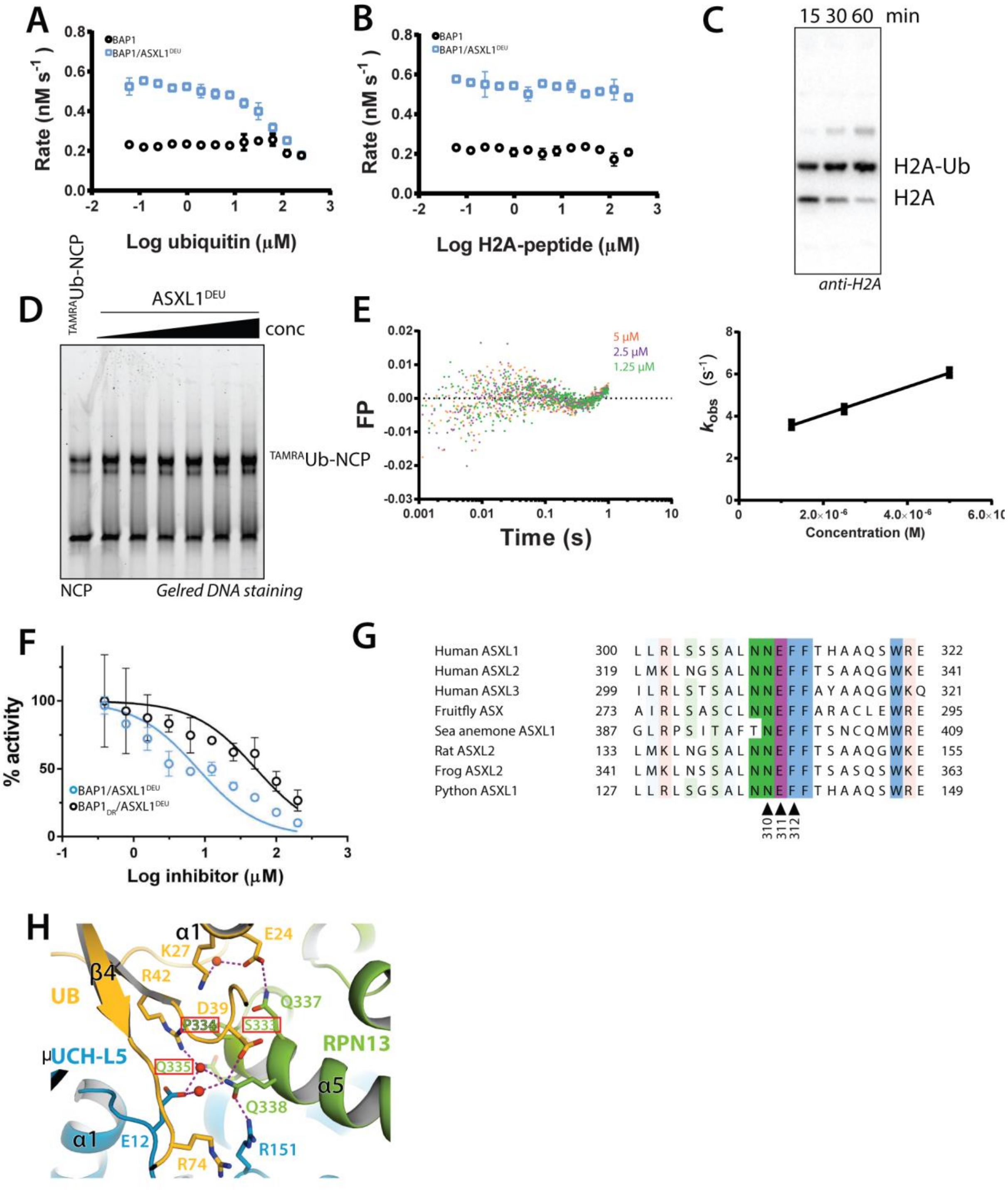

Supplementary Figure 3. Molecular activation mechanism of BAP1 by ASXL1^{DEU}. (a) In Ub-AMC hydrolysis assays, addition of ubiquitin alone does not inhibit BAP1 (black) and mildly inhibits BAP1/ASXL1^{DEU} (blue). **(b)** Tititration of the C-terminal H2A peptide cannot inhibit Ub-AMC hydrolysis of BAP1 (black) nor the complex (blue). **(c)** Anti-H2A western blot of the monoubiquitination of H2A K119 in NCPs with ^{TAMRA}Ub by full length RING1B/BMI1. **(d)** Native gel band shift assay visualized with GelRed DNA staining highlights that ASXL1^{DEU} by itself cannot shift ^{TAMRA}Ub-NCPs (concentration series is 2-fold dilution starting from 15 micromolar). **(e)** Residuals (left) and linear plot (right) for binding of BAP1/ASXL1^{DEU} to ^{TAMRA}Ub-NCPs in stopped-flow fluorescent polarization binding assays gives an approximate Kd of 4 micromolar. BAP1 alone data could not be fitted. **(f)** BAP1$_{DR}$/ASXL1^{DEU} (black) has a higher IC50 than the WT complex (blue) when inhibited by a non-hydrolyzable H2A-K119 ubiquitin conjugate in Ub-AMC assays. **(g)** Multiple sequence alignment shows that the DEUBAD domains in the ASXL family are highly conserved around the "NEF" region. **(h)** In the structure of UCH-L5 (blue) in complex with RPN13^{DEU} (green) and ubiquitin (yellow), the region equivalent to the ASXL1^{DEU} "NEF" region in RPN13^{DEU}(residues in red boxes) contact ubiquitin (pdb 4uel).

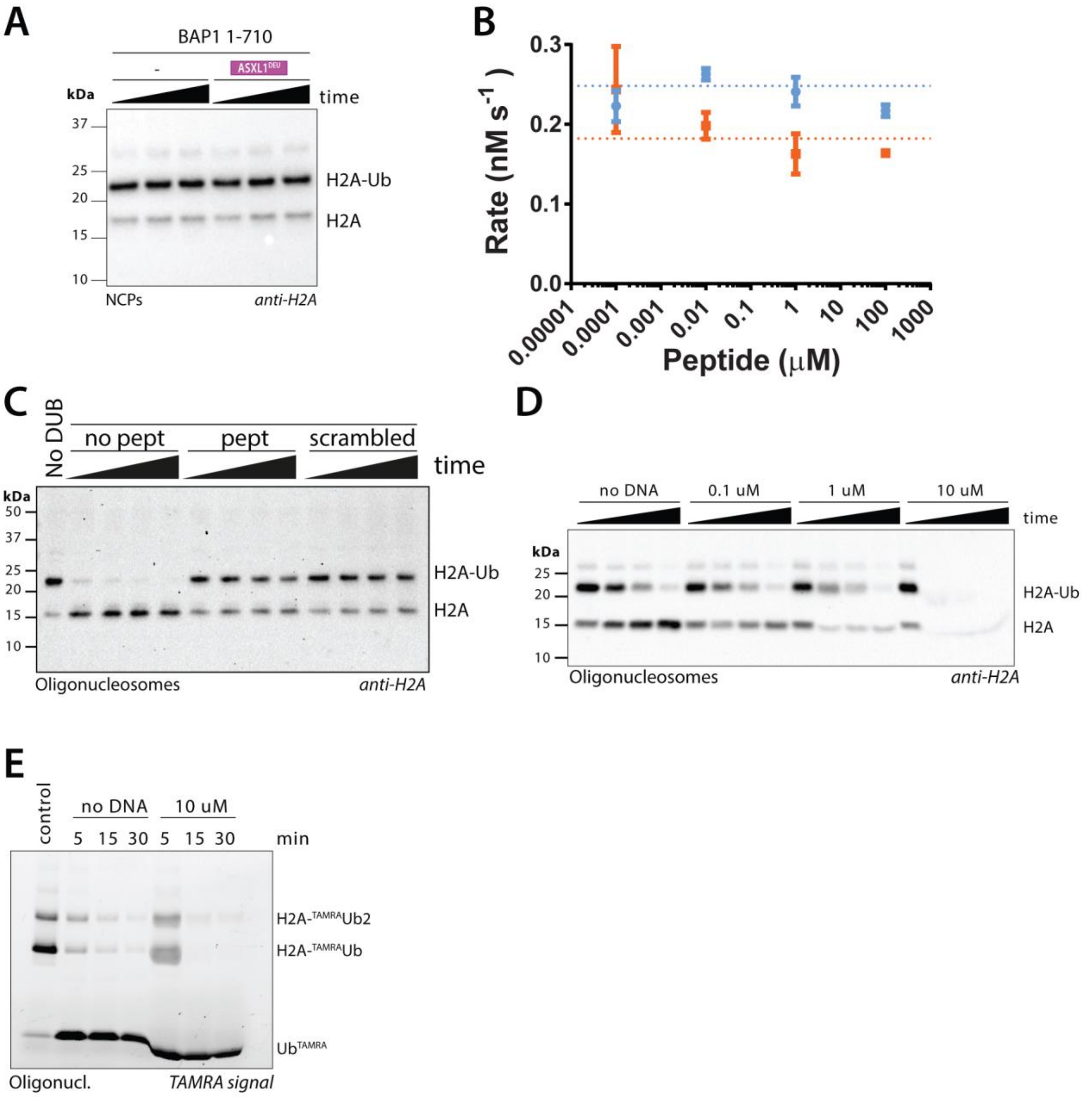

Supplementary Figure 4. The BAP1 CTE is required for nucleosome binding. (a) NCP DUB assay. On NCPs, $BAP1_{1\text{-}710}$ is not active and can also not be activated by the DEUBAD domain of ASXL1 as shown by anti-H2A western blot (time point: 0, 1, 5 min) **(b)** On Ub-AMC, the intrinsic enzymatic activities of full length BAP1 (orange) and $BAP1_{1\text{-}710}$ (blue) are not affected by various concentrations of the BAP1 C-terminal extension peptide. Dotted lines represent the rate in absence of peptide. **(c)** A scrambled peptide of the BAP1 C-terminus is also able to inhibit oligonucleosomal H2A DUB activity of the complex. Time points (7.5, 15, 22.5, 30min). Concentration peptides was 100 µM. **(d)** Anti-H2A blot of a DNA competition assay. BAP1/$ASXL1^{DEU}$ H2A deubiquitinating activity is not inhibited by the presence of 1 µM or 0.1 µM 146 bp alpha satellite DNA. At 10 µM of DNA western blotting problems occurred preventing the efficient transfer of these reactions. This was likely due to the high concentration of DNA in the reaction. **(e)** DUB assay with oligonucleosomal H2A ubiquitinated at K119 with ^{TAMRA}ubiquitin as a substrate, shows that the BAP1/$ASXL1^{DEU}$ complex can deubiquitinate this substrate even in the presence of 10 µM of 146 bp alpha satellite DNA as seen by the increased signal of free ^{TAMRA}Ubiquitin compared to the control.

Supplementary Table 1. Ub-AMC Enzyme kinetics parameters

	k_{cat} (s^{-1})			K_M (μM)			k_{cat}/K_M ($M^{-1}s^{-1}$) *10^{-5}
BAP1	2,5	±	0,2	5,9	±	1,1	4,2
BAP1/ASXL1^{DEU}	1,8	±	0,1	1,3	±	0,2	13,9
BAP1$_{1-710}$	1,6	±	0,1	3,1	±	0,6	5,2
BAP1$_{1-710}$/ASXL1^{DEU}	1,6	±	0,1	0,5	±	0,1	32
BAP1/ASXL1$_{NEF}^{DEU}$	0,6	±	0,03	3,9	±	0,4	1,5
BAP1$_{DR}$/ ASXL1^{DEU}	4,6	±	0,3	2,9	±	0,4	16

Supplementary Table 2. ITC parameters binding experiments

**Cell**	**Syringe**	K_D **(nM)**	ΔH **(kcal/mol)**	$T\Delta S$ **(kcal/mol)**	N
BAP1	ASXL1^{DEU}	18 ± 0.15	-49 ± 0.45	-38	0.37 ± 0.002
BAP1$_{1-710}$	ASXL1^{DEU}	5 ± 0.16	-51 ± 0.37	-40	0.27 ± 0.001
BAP1$_{1-670}$	ASXL1^{DEU}	n/a	n/a	n/a	n/a
BAP1	ASXL1$_{NEF}^{DEU}$	46 ± 0.14	-44 ± 0.54	-34	0.32 ± 0.003

Supplementary Table 3. IC_{50} values for inhibition assays

	IC_{50} **(μM)**		
BAP1	45	±	0,1
BAP1/ASXL1^{DEU}	3,3	±	0,02
BAP1$_{DR}$/ASXL1^{DEU}	52	±	0,1
